# Association of AT1R expression with transplant glomerulopathy and interstitial fibrosis in kidney transplant recipients

**DOI:** 10.3389/fimmu.2026.1882694

**Published:** 2026-07-14

**Authors:** Katarzyna Jakuszko, Piotr Donizy, Agnieszka Sas, Guido Moll, Rusan Catar, Renata Trzeciak-Snopczyńska, Justyna Zachciał, Sławomir Zmonarski, Magdalena Kuriata-Kordek, Agnieszka Hałoń, Maciej Wuczyński, Krzysztof Kujawa, Dariusz Janczak, Mirosław Banasik

**Affiliations:** 1 Department of Nephrology, Transplantation Medicine and Internal Diseases, Institute of Internal Diseases, Wroclaw Medical University, Wroclaw, Poland; 2 University Clinical Hospital in Wroclaw, Wroclaw, Poland; 3 Department of Clinical and Experimental Pathology, Division of General and Experimental Pathology, Wroclaw Medical University, Wroclaw, Poland; 4 Department of Nephrology and Internal Intensive Care Medicine, Charité Universitätsmedizin Berlin, Corporate member of Freie Universität Berlin, Humboldt-Universität zu Berlin, and Berlin Institute of Health (BIH), Berlin, Germany; 5 Julius Wolff Institute (JWI), Charité Universitätsmedizin Berlin, corporate member of Freie Universität Berlin, Humboldt-Universität zu Berlin, and Berlin Institute of Health (BIH), Berlin, Germany; 6 Berlin Institute of Health (BIH) Center and School for Regenerative Therapies (BCRT/BSRT), Charité Universitätsmedizin Berlin, Corporate member of Freie Universität Berlin, Humboldt-Universität zu Berlin, and Berlin Institute of Health (BIH), Berlin, Germany; 7 Department of Clinical and Experimental Pathology, Division of Clinical Pathology, Wroclaw Medical University, Wroclaw, Poland; 8 Statistical Analysis Centre, Wroclaw Medical University, Wroclaw, Poland; 9 Department of Vascular, General and Transplant Surgery, Wroclaw Medical University, Wroclaw, Poland

**Keywords:** allograft survival, anti-AT1R antibodies, AT1R expression, interstitial fibrosis, transplant glomerulopathy

## Abstract

**Background:**

Chronic allograft dysfunction (CAD) remains a major cause of late kidney transplant failure and is driven by both immune and non-immune mechanisms. Transplant glomerulopathy (TG) and interstitial fibrosis (IF) are key histopathological features associated with chronic antibody-mediated injury and long-term graft loss. Experimental and clinical data suggest that angiotensin II type 1 receptor (AT1R) signaling, including activation by anti-AT1R antibodies, may contribute to fibrotic processes within the kidney.

**Methods:**

In this longitudinal study, 77 kidney transplant recipients who underwent indication biopsies within 60 months post-transplantation were analyzed. AT1R expression in allograft tissue was assessed by immunohistochemistry, and serum anti-AT1R antibody levels were measured using ELISA. Histopathological findings and long-term graft outcomes were evaluated over a median follow-up of 126 months.

**Results:**

AT1R expression in tubular epithelium was observed in 44.2% of patients. TG and IF were significantly more frequent in patients with positive AT1R expression compared to those without (24% vs. 7%, p=0.042; 56% vs. 27%, p=0.011, respectively), and IF severity was higher in the AT1R-positive group (p=0.028). Anti-AT1R antibody levels did not differ between groups. In survival analyses, neither AT1R expression nor anti-AT1R antibodies alone were associated with allograft survival. However, the presence of multiple risk factors, including AT1R expression, anti-AT1R antibodies, and histopathological lesions, was associated with significantly worse long-term graft survival.

**Conclusions:**

AT1R expression is associated with the presence and severity of interstitial fibrosis and transplant glomerulopathy in kidney transplant recipients. While AT1R-related markers alone did not predict graft survival, their combination with histopathological abnormalities identifies patients at higher risk of long-term allograft loss. These findings support a potential role of AT1R-mediated pathways in chronic allograft injury.

## 1 Introduction

Kidney transplantation remains the optimal treatment for patients with end-stage renal disease (ESRD), significantly improving both survival and quality of life compared with dialysis. Despite advances in immunosuppressive therapy, particularly targeting T lymphocytes, which have reduced the incidence of acute rejection, long-term graft survival has not improved proportionally. Consequently, late allograft loss remains a major clinical challenge after kidney transplantation (1).

Chronic allograft dysfunction (CAD) is a multifactorial process driven by both immune- and non-immune-mediated mechanisms. The most common contributors include chronic antibody-mediated rejection (AMR), interstitial fibrosis and tubular atrophy (IF/TA), and chronic allograft nephropathy (2). Chronic allograft nephropathy is characterized by progressive fibrosis, vascular injury, and glomerular damage, often resulting from ischemia–reperfusion injury, subclinical or untreated rejection, recurrence or *de novo* glomerulonephritis, and calcineurin inhibitor toxicity. Histopathological features include interstitial fibrosis, arteriolar hyalinosis, and glomerulosclerosis (3). Among these entities, AMR is recognized as a leading cause of late graft failure, with transplant glomerulopathy (TG) representing its key morphological manifestation (4). TG is frequently associated with donor-specific antibodies (DSA), C4d deposition, and peritubular capillaritis (1), and according to Banff criteria, it can be classified into isolated TG, chronic AMR, or chronic active AMR (5, 6).

While anti-human leukocyte antigen (anti-HLA) antibodies are well-established mediators of graft injury, increasing evidence highlights the role of non-HLA antibodies in both acute and chronic allograft damage (7, 8). These antibodies arise from dysregulated B-cell responses and may target G protein–coupled receptors (GPCRs), thereby modulating intracellular signaling pathways. Among them, agonistic autoantibodies directed against the angiotensin II type 1 receptor (AT1R), a key component of the renin–angiotensin system, have gained particular attention (9–11). Elevated levels of anti-AT1R antibodies have been associated with vascular rejection, allograft injury, and graft loss (11–13). Moreover, these antibodies may contribute to the development of post-transplant focal segmental glomerulosclerosis (FSGS), and pre-transplant anti-AT1R levels have been linked to FSGS recurrence (14, 15).

Previous studies from our group demonstrated that AT1R expression in the tubular epithelium of renal transplant biopsies is associated with an increased risk of early graft loss. Specifically, patients with positive AT1R staining had significantly higher one-year post-biopsy graft loss rates compared with those without expression (16). Additionally, elevated anti-AT1R antibody levels were identified as independent risk factors for graft failure (13, 17). Beyond transplantation, increased interstitial AT1R expression has been associated with progressive fibrosis in native kidney diseases, suggesting a potential role in fibrogenesis (18). Taken together, these findings indicate that both AT1R expression and anti-AT1R antibodies may contribute to chronic allograft injury.

Therefore, the aim of this study was to evaluate AT1R expression in kidney allograft biopsies and the presence of anti-AT1R antibodies in kidney transplant recipients, and to assess their association with chronic allograft dysfunction and long-term graft survival.

## 2 Materials and methods

### 2.1 Study population

This longitudinal follow-up study included kidney transplant recipients (KTRs) who underwent indication biopsies within 60 months after transplantation. Initially, 101 patients were enrolled (mean age 44.9 ± 14.6 years; median 46.0 [IQR 32.0-57.0]), at a median of 2.0 months ([IQR 0.0-11.0]; mean 11.6 ± 18.1 months) post-transplantation. Patients were consecutively recruited among those hospitalized for clinically indicated allograft biopsy. Written informed consent was obtained from all participants prior to study inclusion.

During follow-up, 22 patients were lost due to transfer to another transplant center (n=18) or death (n=4), and 2 patients were excluded due to primary non-function of the allograft. The final study population consisted of 77 patients (mean age 43.1 ± 14.1 years; median 44.0 [IQR 32.0-55.0]), evaluated at a median of 6.0 months ([IQR 1.0-24.0]; mean 14.9 ± 19.5 months) after transplantation.

Indications for allograft biopsy included suspected acute rejection (n=5), delayed graft function (n=10), chronic allograft dysfunction (n=23), unexplained deterioration of graft function (n=24), isolated proteinuria (n=1), or combined deterioration of graft function with proteinuria (n=14). The majority of biopsies were performed within the first 6 months post-transplantation (n=45), while the remaining patients were biopsied later (>6 months post-transplantation). None of the patients from the study group were receiving angiotensin receptor blocker (ARB) therapy at the time of allograft biopsy.

Basic demographic characteristics of the final study group are presented in **Table 1**.

**Table 1:** Basic demographic characteristics of the study group (n=77).

Characteristics	≤6 months (n=45)	>6 months (n=32)
Recipients sex (male/female)	34 (76%)/11 (24%)	20 (63%)/12 (38%)
Recipients age (years)	45.2 ± 13.5 (47.0)	40.1 ± 14.7 (41.0)
Donors sex (male/female) Missing data	21 (47%)/13 (29%) 11 (24%)	13 (41%)/11 (34%) 8 (25%)
Donors age (years)	48.4 ± 15.4 (53.0)	47.8 ± 12.2 (45.5)
Cold ischemia time (hours)	21.3 ± 8.8 (24.0)	20.9 ± 9.3 (21.0)
No of HLA mismatches: Total HLA-A HLA-B HLA-DR	3.6 ± 1.1 (4.0) 1.3 ± 0.6 (1.0) 1.3 ± 0.6 (1.0) 0.9 ± 0.6 (1.0)	3.6 ± 1.0 (4.0) 1.4 ± 0.5 (1.0) 1.4 ± 0.6 (1.0) 0.8 ± 0.6 (1.0)
PRA: Last Max	4.8 ± 17.2 (0.0) 14.5 ± 26.1 (3.0)	3.5 ± 11.6 (0.0) 6.5 ± 14.8 (0.0)
Cause of ESRD -glomerulonephritis -hypertension -type 2 diabetes before transplantation -chronic tubulointerstitial nephritis -CAKUT -genetical disorders polycystic kidney disease Alport syndrome -unknown	28 (62%) 3 (7%) 0 (0%) 1 (2%) 3 (7%) 6 (13%) 0 (0%) 4 (9%)	12 (38%) 4 (13%) 2 (6%) 4 (13%) 1 (3%) 3 (9%) 1 (3%) 5 (16%)
Immunosuppressive therapy -glucocorticoid, Tac, MMF -glucocorticoid, Tac, MPS -glucocorticoid, Tac -glucocorticoid, CsA, MMF -glucocorticoid, CsA, MPS -glucocorticoid, CsA -glucocorticoid, Tac/CsA, EVR	26 (58%) 8 (18%) 1 (2%) 6 (13%) 2 (4%) 0 (0%) 2 (4%)	16 (50%) 4 (13%) 4 (13%) 6 (19%) 0 (0%) 1 (3%) 1 (3%)

Data are presented as the number of patients (%) and mean ± SD (median). CAKUT, Congenital Anomalies of the Kidneys and Urinary Tract; CsA, Cyclosporin A; ESRD, End-Stage Renal Disease; EVR, Everolimus; HLA, Human Leukocyte Antigens; MMF, Mycophenolate Mofetil; MPS, Mycophenolate Sodium; PRA, Panel Reactive Antibody; Tac, Tacrolimus; .

### 2.2 Ethics

The study was conducted in accordance with the principles of the Declaration of Helsinki and approved by the Bioethics Committee of Wroclaw Medical University (approval No. KB 224/2023). Written informed consent was obtained from all participants prior to inclusion in the study. All data were anonymized at an early stage of data collection to ensure patient confidentiality.

### 2.3 AT1R immunohistochemical expression analysis

AT1R expression in renal allograft biopsies was assessed by immunohistochemistry, as previously described (16). Briefly, 4 µm-thick paraffin-embedded tissue sections were mounted on silanized slides (Dako, Glostrup, Denmark) and incubated with a mouse monoclonal anti-AT1R antibody (clone sc-81671, 1E10-1A9; dilution 1:100; Santa Cruz Biotechnology, USA).

Immunostaining was evaluated by light microscopy, and AT1R expression was assessed in five anatomical compartments: tubular epithelium, glomeruli, peritubular capillaries, interstitium, and renal blood vessels (small and medium-sized arteries). Expression was graded using a semi-quantitative three-point scale: 0 (no expression), 1 (low expression), and 2 (high expression).

For the purposes of this study, histopathological features of chronic allograft injury were assessed, including the presence of transplant glomerulopathy (TG), focal segmental glomerulosclerosis (FSGS), interstitial fibrosis (IF), and interstitial fibrosis with tubular atrophy (IF/TA). Quantitative parameters included the number and percentage of sclerotic glomeruli, the extent of interstitial fibrosis expressed as a percentage of the affected interstitium, and IF/TA severity graded on a three-point scale.

### 2.4 Anti-AT1R antibodies assay

Blood samples were collected at study enrollment during routine venipuncture using additional tubes. After clot formation, samples were centrifuged at 1500 × g for 10 minutes at room temperature. Serum was separated and stored at −80 °C until analysis.

Serum concentrations of anti-AT1R antibodies were measured using a commercially available enzyme-linked immunosorbent assay (ELISA) kit (CellTrend, Luckenwalde, Germany), according to the manufacturer’s instructions. Briefly, serum samples diluted 1:100 and standards were added to precoated microtiter plates and incubated for 2 hours at 2–8 °C. After washing, bound antibodies were detected using a horseradish peroxidase (HRP)-conjugated anti-human IgG antibody (1:100). Color development was achieved using 3,3′,5,5′-tetramethylbenzidine (TMB) substrate, and absorbance was measured at 450 nm with a reference wavelength of 630 nm.

Antibody concentrations were calculated from standard curves generated from optical density measurements. The assay detection range was 2.5–40.0 U/mL. Based on previous studies, values >10.0 U/mL were considered positive, while values ≤10.0 U/mL were classified as negative (17).

### 2.5 Sample collection and routine parameters assessment

At the time of biopsy, standard clinical parameters were collected, including serum creatinine, estimated glomerular filtration rate (eGFR), and proteinuria. These parameters were also recorded at the last follow-up visit. In patients who required initiation of renal replacement therapy (RRT), the last available laboratory results prior to RRT initiation were used for analysis.

Serum creatinine was measured using routine laboratory methods. Proteinuria was assessed at baseline and during follow-up using a semi-quantitative automated scale with predefined thresholds of 0.1, 0.3, 0.5, 1.0, 3.0, 5.0, and 10.0 g/L.

To ensure consistency across the study period, eGFR was recalculated for all patients using standardized equations based on serum creatinine, age, and sex. Both the Modification of Diet in Renal Disease (MDRD) equation and the Chronic Kidney Disease Epidemiology Collaboration 2021 (CKD-EPI 2021) race-free equation were applied.

### 2.6 Statistical analysis

Descriptive statistics were calculated for all demographic and clinical characteristics, as well as quantitative variables. The normality of data distribution was assessed using the Shapiro–Wilk test. Continuous variables are presented as mean ± standard deviation (SD) for normally distributed data or median with interquartile range (IQR) for non-normally distributed data.

Categorical variables are presented as counts and percentages and were compared using the Pearson chi-square test or Fisher’s exact test, as appropriate (when expected cell counts were ≤5). Group comparisons for continuous variables were performed using the Student’s t-test for normally distributed data or the Mann–Whitney U test for non-normally distributed data.

Cumulative survival was estimated using the Kaplan–Meier method and compared using log-rank tests with pairwise *post hoc* analysis. A p-value <0.05 was considered statistically significant. All statistical analyses were performed using Statistica software (version 12.0; StatSoft).

## 3 Results

### 3.1 AT1R immunohistochemical expression

**Figure 1:**
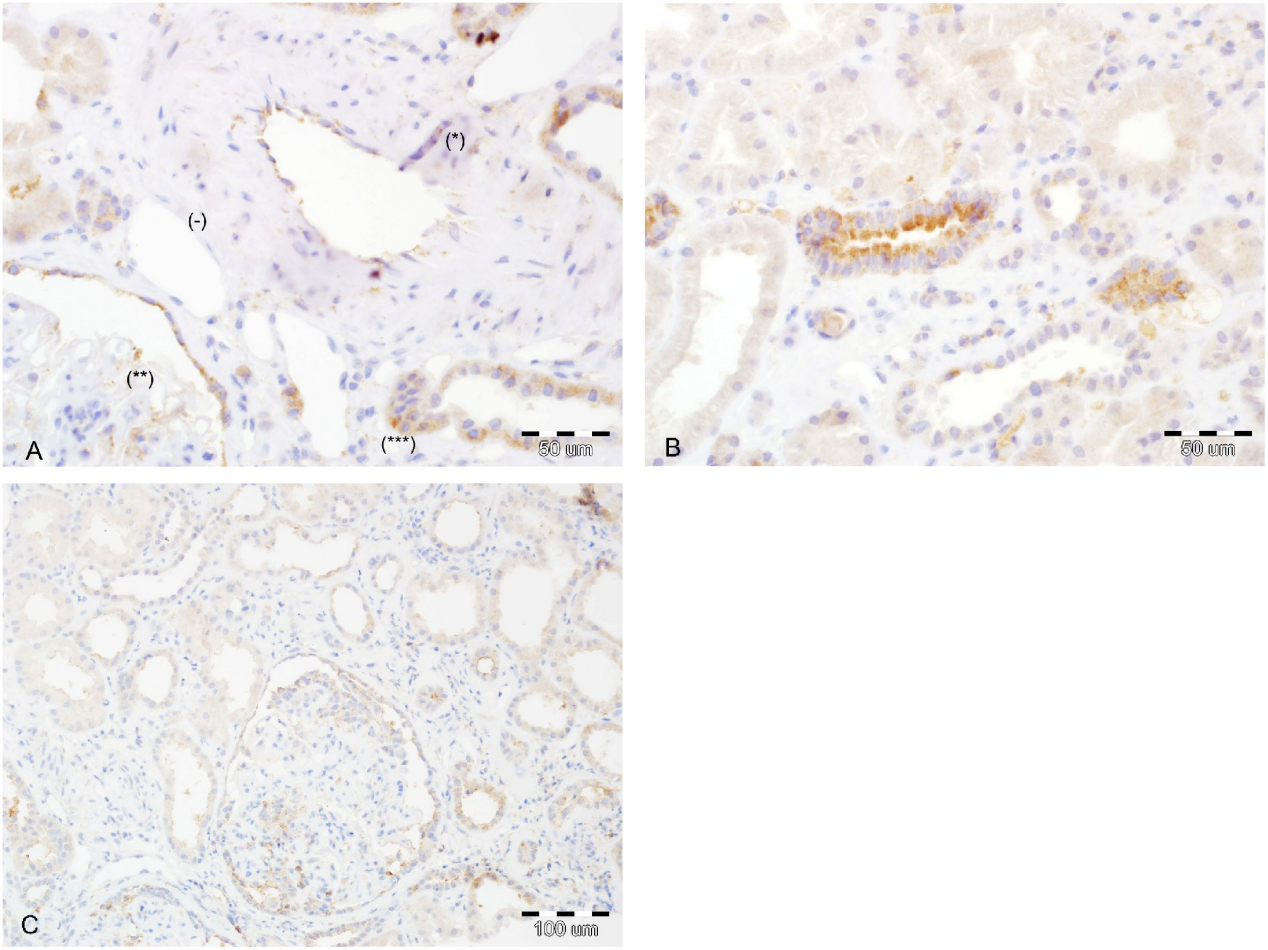
AT1R immunohistochemical expression in renal transplant. **(A)** Positive AT1R expression assessed as 1 in three-point scale in endothelial cells of small vessel (*), glomerulus (**) and tubular epithelium (***). AT1R negative expression assessed as 0 (-). 400x. **(B)** Enhanced (high) expression in tubular epithelium assessed as 2 in three-point scale. 400x. **(C)** Positive AT1R expression assessed as 1 in three-point scale in glomerulus. 200x.

Positive AT1R immunostaining in the tubular epithelium was observed in 34 patients (44.2%), including 26 with low immunoreactivity and 6 with high expression. AT1R expression in other compartments was infrequent: glomerular expression was detected in 3 patients, while expression in peritubular capillaries and the interstitium was observed in only one patient each. In all cases, extratubular expression was accompanied by positive tubular staining. AT1R immunohistochemical expression is presented in **Figure 1**.

### 3.2 Characteristics of the patients

No significant differences were observed between AT1R-positive and AT1R-negative groups with respect to baseline demographic and clinical characteristics, including recipient and donor age, cold ischemia time, number of HLA mismatches, panel reactive antibodies (PRA), serum creatinine, estimated glomerular filtration rate (eGFR), and proteinuria at the time of biopsy and at the last follow-up. Detailed characteristics of the study groups are presented in **Table 2**.

**Table 2:** Demographic and clinical characteristics of the study groups (n=77).

Characteristics	AT1R positive (n=34)	AT1R negative (n=43)	P value
Recipients sex (male/female)	26 (76%)/8 (24%)	28 (65%)/15 (35%)	0.279 *
Recipients age (years)	45.0 (33.0-55.0)	44.0 (32.0-56.0)	0.914 ***
Donors sex (male/female) Missing data	19 (56%)/8 (24%) 7 (21%)	15 (35%)/16 (37%) 12 (28%)	0.178 *
Donors age (years)	54.5 (39.0-60.5)	50.0 (37.5-58.0)	0.407 ***
Cold ischemia time (hours)	21.3 ± 7.9	21.0 ± 9.9	0.899 **
No of HLA mismatches: Total HLA-A HLA-B HLA-DR	3.0 (3.0-4.0) 1.0 (1.0-2.0) 1.0 (1.0-2.0) 1.0 (0.0-1.0)	4.0 (3.0-5.0) 1.0 (1.0-2.0) 1.0 (1.0-2.0) 1.0 (0.0-1.0)	0.379 *** 0.782 *** 0.825 *** 0.270 ***
PRA: Last Max	0.0 (0.0-0.0) 0.0 (0.0-37.0)	0.0 (0.0-0.0) 0.0 (0.0-4.0)	0.476 *** 0.323 ***
Laboratory results at time of biopsy: serum creatinine eGFR MDRD eGFR CKD-EPI 2021 proteinuria	2.12 (1.80-2.82) 31.2 ± 10.7 32.9 (24.3-38.0) 35.1 ± 12.6 37.5 (27.0-44.0) 0.1 (0.0-1.0)	2.12 (1.79-2.90) 31.6 ± 12.4 33.0 (21.8-39.6) 35.6 ± 14.7 37.0 (24.0-45.0) 0.1 (0.0-0.5)	0.918 *** 0.880 ** 0.858 ** 0.510 ***
Laboratory results at time of last observation: serum creatinine eGFR MDRD eGFR CKD-EPI 2021 proteinuria	2.62 (1.63-5.32) 21.0 (11.3-38.9) 23.0 (12.0-44.0) 0.3 (0.0-1.0)	2.51 (1.47-5.01) 27.6 (9.7-48.4) 30.0 (11.0-53.0) 0.1 (0.0-1.0)	0.550 *** 0.667 *** 0.667 *** 0.991 ***
RAS blockade received after allograft biopsy: ACEI ARB	3 (8.8%) 3 (8.8%) 0 (0.0%)	6 (13.9%) 5 (11.6%) 1 (2.3%)	0.372 #

Data are presented as n (% of pts) and mean ± SD (for normally distributed numerical values) or median (IQR) for not normally distributed data; p-values from Pearson’s chi-squared test (*); Fisher’s exact test (#); t-test (**) or non-parametric Mann-Whitney test (***); ACEI: angiotensin-converting enzyme inhibitor; ARB, angiotensin receptor blocker; AT1R, Angiotensin II Type 1 Receptor; CKD-EPI 2021, Chronic Kidney Disease Epidemiology race-free 2021 equation; eGFR, estimated Glomerular Filtration Rate; HLA, Human Leukocyte Antigens; MDRD, Modification of Diet in Renal Disease; PRA, Panel Reactive Antibody; RAS, renin-angiotensin system.

### 3.3 Anti-AT1R antibodies and histopathological changes in patients with positive or negative AT1R expression in tubular epithelium.

Histopathological changes characteristic of chronic allograft injury were more frequently observed in patients with positive AT1R expression. TG and IF were identified in 8 (24%) and 18 (56%) patients in the AT1R-positive group, compared with 3 (7%) and 11 (27%) patients in the AT1R-negative group (p=0.042 and p=0.011, respectively).

**Figure 2:**
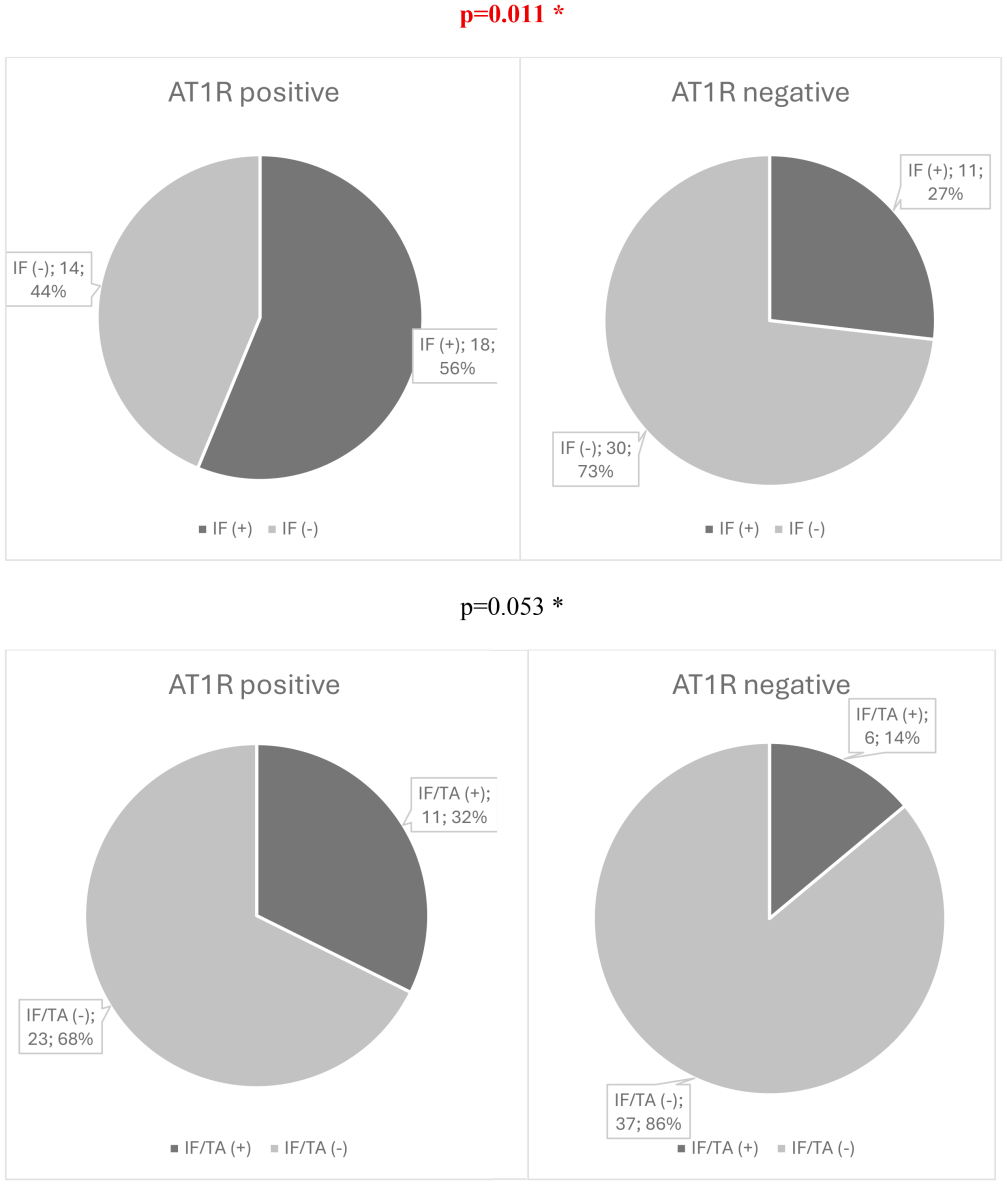
Histopathological changes (IF and IF/TA) in patients with positive and negative AT1R expression in tubular epithelium. AT1R, angiotensin II type 1 receptor; IF, interstitial fibrosis; IF/TA, interstitial fibrosis and tubular atrophy; p-values from Pearson’s chi-squared test (*).

**Figure 3:**
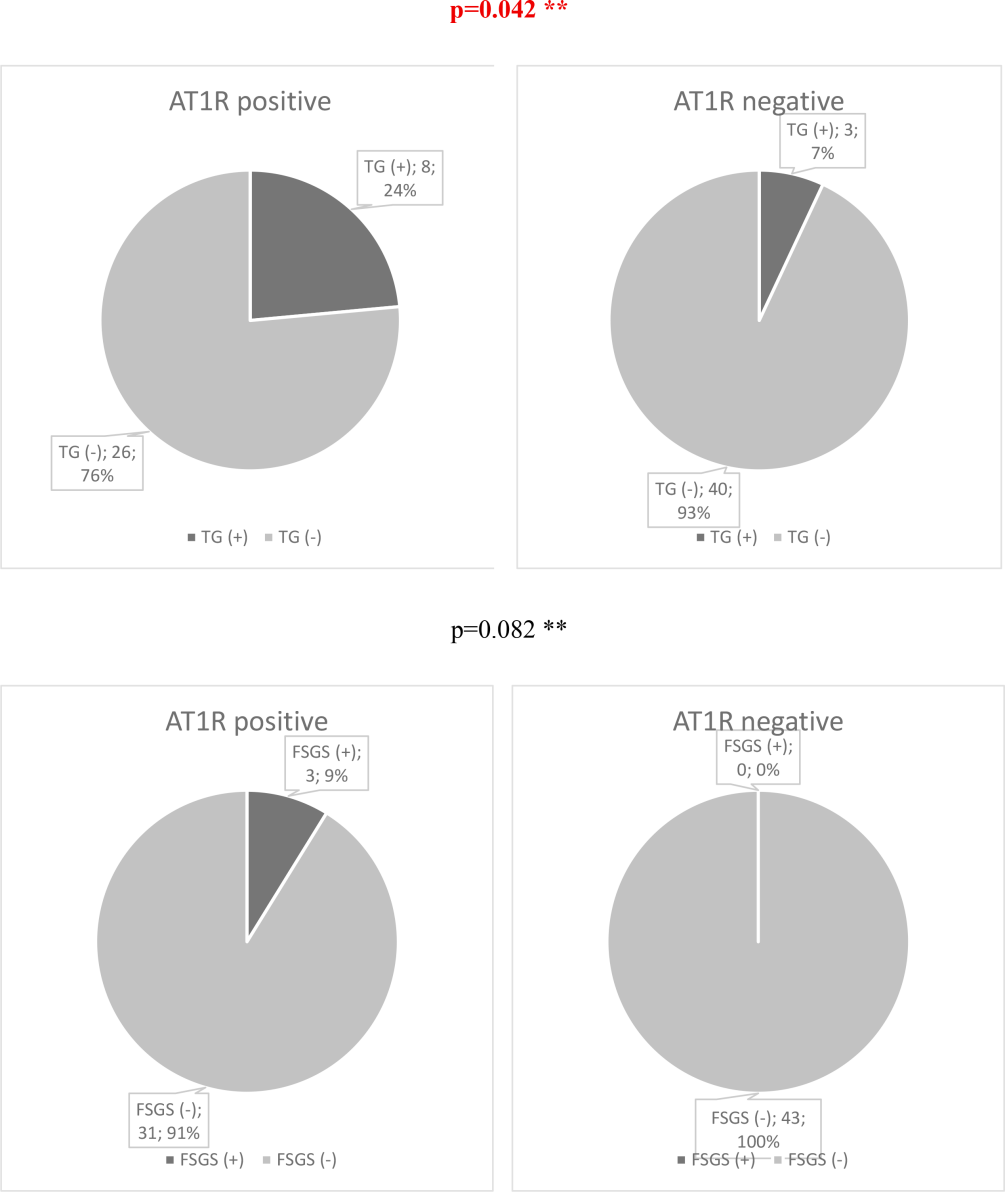
Histopathological changes (TG and FSGS) in patients with positive and negative AT1R expression in tubular epithelium. AT1R, angiotensin II type 1 receptor; FSGS, focal segmental glomerulosclerosis; TG, transplant glomerulopathy; p-values from Fisher’s exact test (**).

A detailed comparison of histopathological findings according to AT1R expression in the tubular epithelium is presented in **Figures 2** and **3**.

The severity of IF was higher in AT1R positive group (p=0.028). There were no differences in number and percentage of glomerulosclerosis, IF/TA severity and serum anti-AT1R antibodies concentrations in patients with positive and negative AT1R expression (**Table 3**).

**Table 3:** Histopathological changes and anti-AT1R antibodies in AT1R positive and negative patients (n=77).

Assessed parameter	AT1R positive (n=34)	AT1R negative (n=43)	P value
Histopathological changes:			
TG [yes] TG [no]	8 (24%) 26 (76%)	3 (7%) 40 (93%)	0.042 **
FSGS [yes] FSGS [no]	3 (9%) 31 (91%)	0 (0%) 43 (100%)	0.082 **
glomerulosclerosis [n]	0.0 (0.0-2.5)	0.0 (0.0-2.0)	0.947 ***
glomerulosclerosis [%]	0.0 (0.0-14.8)	0.0 (0.0-11.1)	0.960 ***
IF [yes] IF [no]	18 (56%) 14 (44%)	11 (27%) 30 (73%)	0.011 *
IF [%]	5.0 (0.0-13.5)	0.0 (0.0-5.0)	0.028 ***
IF/TA [yes] IF/TA [no]	11 (32%) 23 (68%)	6 (14%) 37 (86%)	0.053 *
IF/TA severity [3-step scale]	0.0 (0.0-1.0)	0.0 (0.0-0.0)	0.165 ***
Acute AMR [yes] Acute AMR [no]	5 (14.7%) 29 (85.3%)	3 (6.9%) 40 (93.1%)	0.233 **
Laboratory results at time of biopsy:			
anti-AT1R [U/mL]	7.59 (2.85-11.70)	7.70 (3.44-10.63)	0.991 ***
anti-AT1R positive anti-AT1R negative	10 (31%) 22 (69%)	12 (29%) 29 (71%)	0.855 *

Data are presented as n (% of pts) and median (IQR) as all data were not normally distributed; p-values from Pearson’s chi-squared test (*); Fisher’s exact test (**) or non-parametric Mann-Whitney test (***); AT1R, Angiotensin II Type 1 Receptor; FSGS, focal segmental glomerulosclerosis; IF, Interstitial Fibrosis; IF/TA, Interstitial Fibrosis and Tubular Atrophy; TG – transplant glomerulopathy;.

We identified only 8 cases of acute AMR in this study cohort – 5/34 (14.7%) in AT1R positive patients and 3/43 (6.9%) in AT1R negative patients (p=0.233) (**Table 3**). Moreover acute AMR was observed in 4/22 (18.2%) with positive anti-AT1R and in 4/51 (7.8%) with negative anti-AT1R (p=0.185) (Data not shown). Acute AMR was treated with methylprednisolone intravenously in total dose: 1500 mg (n=4), 1000 mg (n=1), 2250 mg (n=1) or 2500 mg (n=2); plasmapheresis (n=3); intravenous immunoglobulin (n=4); anti-thymocyte globulin (n=2); rituximab 500 mg (n=1). No differences were found between patients with acute AMR regarding allograft function, however eGFR at the time of biopsy was lower with marginal statistical significance (eGFR MDRD p=0.060 and eGFR CKD-EPI p=0.088). Three patients with acute AMR had allograft loss after 15, 35 and 164 months post-transplantation.

### 3.4 Allograft survival

Allograft survival was defined as the absence of the need for RRT at the end of the observation period. The median follow-up duration among patients with preserved allograft function (n=43) was 126.0 months (IQR 111.0–144.0).

The overall 5- and 10-year allograft survival rates during the study period were 70.1% and 57.8%, respectively. When calculated from the time of transplantation, the 5-, 10-, and 15-year allograft survival rates were 81.8%, 60.2%, and 48.2%, respectively.

Allograft loss occurred in 34 patients. Renal replacement therapy was initiated after a median of 32.0 months post-biopsy (IQR 10.0–69.0) or 62.5 months post-transplantation (IQR 35.0–90.0).

Receiver operating characteristic (ROC) curve analysis was performed to evaluate the association between AT1R expression intensity, anti-AT1R antibody levels, serum creatinine, eGFR (MDRD), and eGFR (CKD-EPI 2021) at the time of biopsy and the risk of allograft loss. The area under the curve (AUC), optimal cut-off values, and 95% confidence intervals (CI) are presented in **Table 4**.

**Table 4:** AUC, cut-off values and 95% CI for allograft loss.

Assessed parameter	AUC	Cut-off	95% CI	P
AT1R expression	0.517	2.0	0.385-0.649	0.799
Anti-AT1R	0.494	12.3	0.355-0.634	0.936
Serum creatinine	0.584	2.06	0.454-0.715	0.205
eGFR MDRD	0.599	34.4	0.466-0.732	0.143
eGFR CKD-EPI 2021	0.605	39.0	0.472-0.737	0.121

AT1R, Angiotensin II Type 1 Receptor; AUC, Area under the curve; CI, confidence interval; CKD-EPI 2021, Chronic Kidney Disease Epidemiology race-free 2021 equation; eGFR, estimated Glomerular Filtration Rate; MDRD, Modification of Diet in Renal Disease.

The Kaplan-Meier analysis of single factors, including AT1R expression or anti-AT1R, didn’t detect any statistically significant impact on long-term allograft survival (p=0.832, p=0.438, respectively) (Data not shown). Therefore we decided to analyze the combined effects of both AT1R expression and anti-AT1R, however allograft survival also didn’t differ between groups with both factors positive and both negative (p=0.270) and between three groups: both factors positive, one factor positive (AT1R+ and anti-AT1R- or AT1R- and anti-AT1R+) and both negative (p=0.419) (Data not shown).

**Figure 4:**
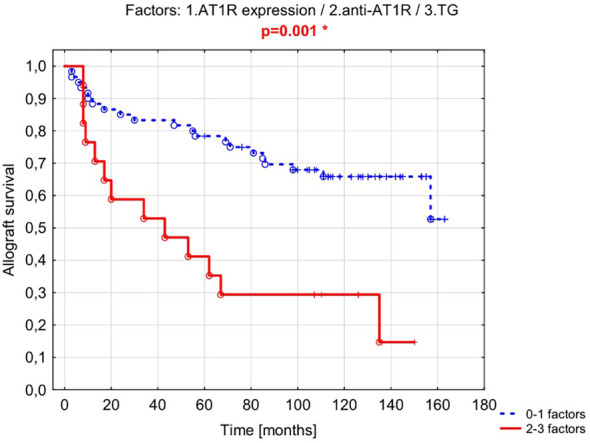
Kaplan-Meier allograft survival curves in relation to presence of AT1R expression, anti-AT1R and/or TG. p-value from global log-rank test (*).

**Figure 5:**
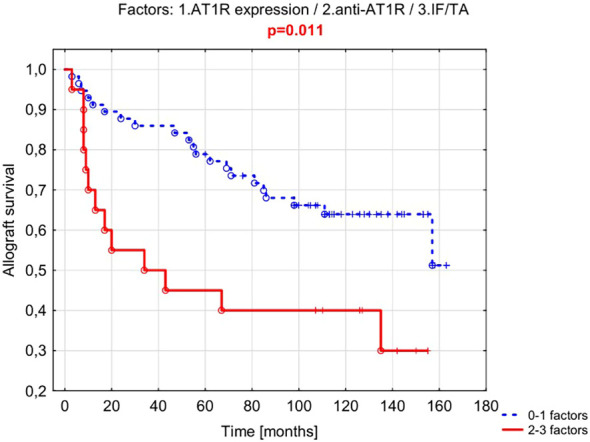
Kaplan-Meier allograft survival curves in relation to presence of AT1R expression, anti-AT1R and/or IF/TA. p-value from global log-rank test (*).

**Figure 6:**
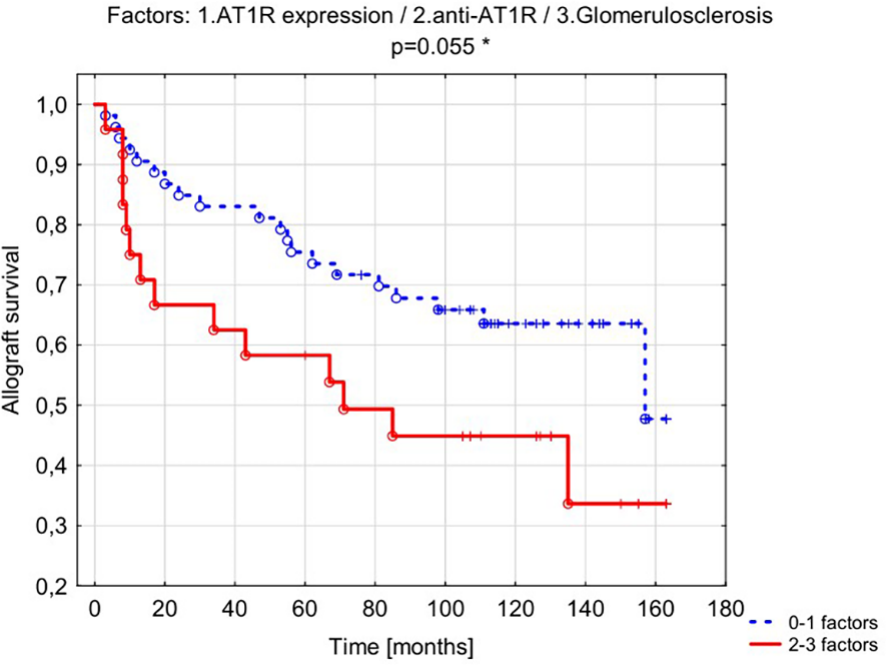
Kaplan-Meier allograft survival curves in relation to presence of AT1R expression, anti-AT1R and/or glomerulosclerosis. p-value from global log-rank test (*).

**Figure 7:**
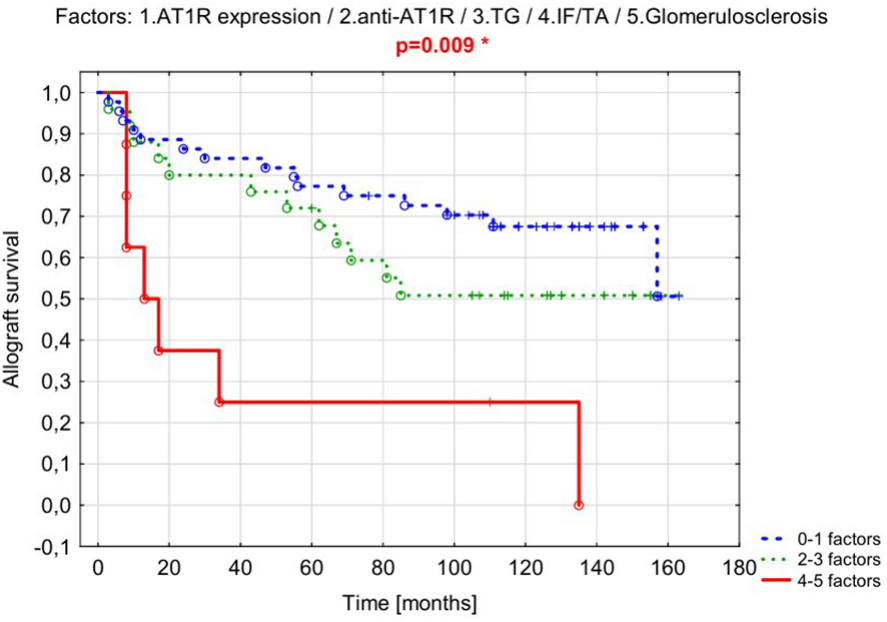
Kaplan-Meier allograft survival curves in relation to presence of AT1R expression, anti-AT1R, TG, IF/TA and/or glomerulosclerosis. p-values from global log-rank test (*) or pairwise *post-hoc* log-rank tests (**).

Finally, allograft survival rates were analyzed according to presence of AT1R expression, anti-AT1R and one of the histopathological changes, including TG, IF/TA, and glomerulosclerosis. Interestingly, the worst allograft survival was observed in patients with 2-3 (in analysis including 3 factors) or 4–5 factors (in analysis including all 5 factors). All data including pairwise *post-hoc* log-rank tests are presented in **Figures 4**–**7**.

Importantly, neither AT1R expression nor anti-AT1R antibodies alone were associated with graft survival, whereas their combination with histopathological lesions identified a subgroup of patients with significantly worse long-term outcomes.

## 4 Discussion

The present study is among the first to systematically evaluate AT1R expression in kidney allograft biopsies in conjunction with circulating anti-AT1R antibodies in the context of chronic allograft dysfunction and long-term graft survival.

Previous studies from our group demonstrated that AT1R expression in the tubular epithelium of renal allograft biopsies is associated with an increased risk of early graft dysfunction and loss. In particular, patients with positive AT1R staining had significantly higher one-year post-biopsy graft loss rates compared with those without expression (35.7% vs. 14.5%) (16). Furthermore, the coexistence of AT1R expression and anti-AT1R antibodies was associated with a markedly increased risk of early graft loss, with one-, two-, and three-year post-biopsy graft loss rates of 37%, 43%, and 50%, respectively, compared with 10%, 17%, and 21% in patients without these markers (17).

Additionally in another study univariate and multivariate analyses of risk factors for acute rejection occurrence showed that anti-AT1R antibodies before transplantation are an independent risk factor for acute rejection (19). The studies from our group showed already that positive AT1R staining in the microcirculation (glomeruli and peritubular capillaries) was found in patients with active AMR (17). Furthermore, patients with microvascular inflammation (MVI), which is a subcategory of AMR, had higher prevalence of anti-AT1R and higher frequency of C4d positivity compared to patients without MVI (20). Due to the limited sample size and only 8 cases of acute AMR in the study group, we are aware of the probability of a Type II error and the need of future studies including the broader population of kidney transplant recipients that could evaluate the association between acute AMR and AT1R expression or circulating anti-AT1R antibodies.

The extended follow-up in the present study (median 126 months) enabled the assessment of long-term graft outcomes. Anti-AT1R antibodies have previously been linked to vascular inflammation, endothelial injury, and progressive deterioration of graft function, all of which may contribute to long-term graft loss (16, 21, 22). In our cohort, the 5- and 10-year allograft survival rates were 70.1% and 57.8%, respectively, while the 10-year survival rate calculated from transplantation was 60.2%, comparable to previously reported outcomes from large transplant centers (23).

Given the multifactorial nature of long-term graft survival, we evaluated outcomes in relation to combined risk factors, including AT1R expression, anti-AT1R antibodies, and histopathological changes such as TG, IF/TA, and glomerulosclerosis. Notably, the poorest graft survival was observed in patients with multiple coexisting risk factors, particularly when both AT1R expression and anti-AT1R antibodies were present. This finding supports a potential cumulative or synergistic effect of these factors in chronic allograft injury.

While several biomarkers have been proposed to predict allograft dysfunction, their utility in forecasting long-term outcomes remains limited (24, 25). Large cohort studies incorporating both indication and surveillance biopsies have identified key predictors of graft loss, including baseline graft function, proteinuria, donor-specific HLA antibodies, and histopathological features such as IF/TA, microvascular injury, inflammation, and tubulitis (2, 26). In this context, our findings suggest that AT1R-related pathways may represent an additional component of the complex network contributing to chronic allograft damage. Interestingly, severe chronic rejection and transplant glomerulopathy can develop without classical donor-specific HLA antibodies. Current transplant immunology guidelines increasingly emphasize post-transplant screening for non-HLA targets. Other than anti-AT1R non-HLA antibodies that have been implicated in chronic rejection and allograft injury, include antibodies against MHC class I chain-related antigens A and B (MICA/MICB) and antibodies against endothelin-1 Type A Receptor (anti-ETAR), which may contribute to chronic alloimmune injury through complementary or overlapping mechanisms (27, 28).

Transplant glomerulopathy, a hallmark of chronic antibody-mediated rejection, was observed more frequently in patients with positive AT1R expression in our study. TG has been consistently associated with poor graft outcomes, irrespective of Banff classification categories, although the severity of chronic injury appears to be a stronger determinant of prognosis than diagnostic subclassification alone (1). Similarly, interstitial fibrosis, a key feature of chronic allograft injury, was more prevalent and more severe in AT1R-positive patients in our cohort. These findings are consistent with previous observations linking AT1R expression to fibrotic processes in both native and transplanted kidneys (18).

The potential mechanistic link between AT1R activation and fibrosis is supported by data from systemic diseases. Unlike angiotensin II, which induces transient and tightly regulated receptor activation, agonistic anti-AT1R autoantibodies can lead to sustained receptor signaling (9, 10). In systemic sclerosis, these antibodies have been implicated in promoting inflammation, angiogenesis, and fibrosis (29). Similarly, in lupus nephritis, anti-AT1R antibodies have been associated with microvascular injury (30).

Taken together, these findings suggest that AT1R expression and anti-AT1R antibodies may contribute to chronic allograft injury, particularly in relation to fibrotic remodeling and microvascular damage. However, their impact on long-term graft survival appears to depend on the coexistence of additional clinical and histopathological risk factors, highlighting the multifactorial nature of chronic allograft dysfunction.

These findings support a potential role of AT1R-related pathways as part of a multifactorial network contributing to chronic allograft injury.

## 5 Conclusions and limitations

To our knowledge, this is one of the first studies to systematically evaluate AT1R expression in kidney allograft biopsies in conjunction with circulating anti-AT1R antibodies in relation to chronic allograft dysfunction and long-term (10-year) graft survival.

Our findings demonstrate that AT1R expression is associated with the presence of transplant glomerulopathy and interstitial fibrosis in kidney transplant recipients. Although the underlying mechanisms were not investigated in this study, AT1R activation may result from both its physiological ligand, angiotensin II, and agonistic anti-AT1R antibodies. Notably, serum anti-AT1R antibody levels did not differ between patients with positive and negative AT1R expression; however, this does not exclude the possibility of antibody sequestration within allograft tissue. Prolonged receptor activation by agonistic antibodies may amplify AT1R signaling beyond that induced by endogenous ligands.

Importantly, neither AT1R expression nor anti-AT1R antibodies alone were associated with long-term graft survival, whereas their combination with histopathological lesions identified a subgroup of patients at higher risk of allograft loss.

These observations highlight the need for further research into AT1R-mediated pathways and their potential as therapeutic targets. In this context, non-immunosuppressive agents such as sparsentan, an AT1R antagonist, have shown efficacy in reducing proteinuria in kidney transplant recipients with recurrent IgA nephropathy or focal segmental glomerulosclerosis (31). Given the association between AT1R activation and fibrotic injury, the therapeutic application of such agents may extend beyond these indications and warrants further investigation.

This study has several limitations that should be acknowledged. First, it was conducted at a single center, which may limit the generalizability of the findings and contributed to a relatively small sample size. Second, the study population consisted exclusively of patients undergoing indication biopsies, which may introduce selection bias and limit applicability to the broader population of kidney transplant recipients. Third, data on donor-specific antibodies and other established immunological risk factors were not included, which may confound the interpretation of the observed associations. Fourth, the observational design of the study precludes causal inference between AT1R expression and histopathological changes. Fifth, no multivariable analysis was performed due to the limited sample size. Finally, anti-AT1R antibody levels were assessed only in serum, and their potential deposition within allograft tissue was not evaluated.

There is emerging evidence suggesting that ARB therapy may have a protective effect in patients with AT1R-mediated allograft injury. Previous studies demonstrated that renin-angiotensin system (RAS) blockade therapy reduced proteinuria, a marker of kidney damage, and stabilized blood pressure, which are critical for maintaining allograft function. Moreover angiotensin-converting enzyme inhibitor (ACEI) or ARB therapy reduced IF/TA progression and delayed rejection relative to reduced tacrolimus exposure without RAS blockade (32). None of the patients from the study group were receiving ARB therapy at the time of allograft biopsy, therefore the possible confounding effect of the treatment on AT1R expression was excluded. Although observational and cohort studies often associate RAS blockade with lower all-cause mortality and allograft loss, randomized controlled trials have not confirmed the evidence on allograft preservation (33). The RAS blockade therapy was used with caution in our cohort of patients, as only 9 patients (11.6%) in the study group were receiving RAS blockade during the observation period. The lack of evaluation of the effect of ACEI or ARB treatment on allograft function or progression of chronic allograft injury may be additional important study limitation.

Nevertheless, mentioned limitations do not diminish the significance of the findings that AT1R expression is associated with the presence of transplant glomerulopathy and interstitial fibrosis in kidney transplant recipients.

The main strength of this study is the long-term follow-up period. Future studies should focus on elucidating the mechanisms of AT1R activation and its role in chronic allograft injury. Improved risk stratification of kidney transplant recipients may be achieved through the integration of histopathological assessment with novel biomarkers such as AT1R expression, potentially enabling earlier identification of patients at risk of graft loss and facilitating targeted therapeutic interventions.

## Data Availability

The raw data supporting the conclusions of this article will be made available by the authors, without undue reservation.
